# Optic Perineuritis and Its Association With Autoimmune Diseases

**DOI:** 10.3389/fneur.2020.627077

**Published:** 2021-01-29

**Authors:** Hongyang Li, Hang Zhou, Jiao Sun, Huihui Wang, Yanling Wang, Zhenchang Wang, Jing Li

**Affiliations:** ^1^Department of Ophthalmology, Beijing Friendship Hospital, Capital Medical University, Beijing, China; ^2^Department of Rheumatology, Beijing Friendship Hospital, Capital Medical University, Beijing, China; ^3^Department of Radiology, Beijing Friendship Hospital, Capital Medical University, Beijing, China

**Keywords:** optic perineuritis, autoimmune diseases, neuromyelitis optica spectrum disorders, multiple sclerosis, optic neuritis

## Abstract

**Background:** Optic perineuritis (OPN) is a special optic neuropathy that has a distinct etiology from neuromyelitis optica spectrum disorders (NMOSDs) or multiple sclerosis (MS)-related optic neuritis (ON). The mechanisms of how this inflammation developed and invaded the nerve sheath remain unknown. This study is aimed to analyze the etiology and different clinical characteristics of OPN in a Chinese patient population.

**Methods:** Neuro-ophthalmological examination, orbit magnetic resonance imaging (MRI) and a series of blood samples were used in this retrospective observational cohort study to compare characteristics of OPN with idiopathic demyelination optic neuritis (IDON).

**Results:** Forty-four OPN cases (74 eyes) and 61 IDON cases (78 eyes) were analyzed. OPN cases included 33 cases (59 eyes) were associated with specific autoimmune diseases, 10 cases (13 eyes) were associated with infection diseases, 1 case was idiopathic disease. The causes of OPN with CTD were Graves' disease, Immunoglobulin G4-related disease (IgG-4 RD), granulomatosis with polyangiitis (GAP), systemic lupus erythematosus (SLE), Sarcoidosis, Rheumatoid arthritis, scleroderma, Behcet's disease, and gout. All patients received orbital MRI. Overall, 33 cases showed orbit fat infiltration. Specifically, nine cases with IgG-4 RD showed trigeminal nerve branch involvement, 12 cases with Graves' disease showed extraocular muscle belly enlargement, and 4 cases with GAP showed pterygopalatine fossa pseudotumor. Compared to IDON patients, OPN patients were older (*p* = 0.004) and more likely bilateral involvement 26 (78.79%) patients had bilateral involvement in OPN group vs. 17 (27.87%) in the IDON group (*p* < 0.001). Visual acuity scores using LogMAR testing was better in OPN patients compared to those with IDON, 0.55 ± 0.91 vs. 1.19 ± 1.24 (*p* < 0.001). Other ophthalmologic findings unique to the OPN group include 11 (33.33%) cases of ptosis, nine (27.27%) cases of diplopia, and 10 (30.30%) cases of exophthalmos, compared to zero cases of these conditions in the IDON group. Eight (13.11%) IDON patients also had multiple sclerosis (MS) and 7 (11.48%) patients had neuromyelitis which was significantly more than the zero patients in OPN group (*p* = 0.04).

**Conclusions:** OPN had distinct etiologies and clinical characteristics from IDON and is more often associated with autoimmune diseases. Using OPN characteristics to diagnose autoimmune diseases should prove useful for clinicians when presented with patients that have multiorgan dysfunction that include ophthalmologic findings.

## Introduction

Optic perineuritis (OPN) is a rare form of orbital inflammatory disease targeting the optic nerve sheath (1). OPN usually presents with minor visual impairment, optic disc edema, and visual field abnormalities that include arcuate defects and peripheral island defects (2). It is very important to identify etiology and clinical characteristics of patients with OPN, and it will affect the treatment or prognosis.

OPN mimics optic neuritis but is distinguished by the classical optic nerve sheath (ONS) enhancement observed on MRI (3). OPN has long been considered an idiopathic inflammatory disease. However, recent reports suggest it may be related to other inflammatory and infectious diseases (4), yet most studies are case reports and lack a detailed analysis of OPN pathogenesis and clinical features. OPN is a special optic neuropathy that has a distinct etiology from neuromyelitis optica spectrum disorders (NMOSDs) or multiple sclerosis (MS)-related optic neuritis (ON). Prior studies often found OPN associated with rheumatic connective tissue diseases (CTDs) (5), yet its pathogenic mechanisms are still unclear. OPN differs from idiopathic demyelination optic neuritis (IDON) which is caused by demyelinating of the optic nerve due to inflammation (6). As we know, it is very important for the treatment, because different treatments for the IDON and other autoimmune diseases. Optic nerve sheath biopsy from a prior study showed, thickening of the perioptic meninges and the pia mater due to fibrosis and inflammatory infiltration, without vasculitis or granulomas, yet mechanisms of how this inflammation developed and invaded the nerve sheath remain unknown (1). Compared with prevision studies, the definition of the disease, its frequency, and the importance of differentiating it from other diseases based on different treatment or prognosis should be reconsidered.

In this study of 44 Chinese patients with OPN, we examine the most common etiologies and using patient imaging data, try to make it different from other diseases based on different treatment or prognosis.

## Materials and Methods

### Patients

Forty-four patients with OPN were recruited from the ophthalmology department of Beijing Friendship Hospital (BFH) at Capital Medical University, in Beijing, China. This is a retrospective cohort study and ocular lesions was the first symptom. Patient recruitment took place from September 2015 to November 2018, and patients meeting the inclusion criteria were offered participation in the study, which included consultation and follow-up outpatient visits. Inclusion criteria were contrast enhancement surrounding the intra-orbital optic nerve and at least one of the following clinical symptoms: (1) reduction of visual acuity, (2) impairment of visual field, or (3) eye pain (1, 2). Patient follow-up varied, lasting from 6 to 36 months. Exclusion: MRI contrast enhancement or T2 lesions of the intra-orbital optic nerve. The 61 optic neuritis were recruitment took place from the September 2015 to May 2019. Inclusion criteria were: (1) MRI contrast enhancement or T2 lesions of the intra-orbital optic nerve. (2) Acute loss of visual acuity or visual field, with or without eye pain. (3) At least one of the following abnormalities: relative afferent pupillary defect, a nerve fiber bundle visual field defect, abnormal visual evoked potential. Exclusion: exclude ischemia, congenital hereditary disease, toxic disease and causes of oppression; intraocular pressure of 21 mmHg or higher; systemic conditions that could affect the visual system; a history of ocular trauma or concomitant ocular diseases; including a history of media opacification, ocular pathologies affecting the cornea, lens, retinal disease, glaucoma, or laser therapy; retina diseases.

### Neuro-Ophthalmological Examination

Ophthalmic examinations included slit lamp examination, pupillary reaction testing, non-contact intraocular pressure examination, and fundus examination by senior neuro-ophthalmologists. Visual acuity was examined using the standard table of vision logarithms at a distance 5 m. Those unable to read any letters at 1 m were further examined using finger counts, hand movements, or light perception. Visual field testing was performed using a Humphrey field Analyzer (30-2 SITA, Humphrey 750i, Zeiss, Germany).

Orbital MRI was performed in all patients and evaluated using MRI-3.0T (TW1WSPEED HDXT, GE, USA). Scanning sequence and parameters included coronal T1-weighted Fast spin-echo (TR = 660 ms; TE = 11.1 ms, matrix size = 256 × 256 mm, FOV = 18 × 18 cm, slice thickness = 3.0 mm) and Gd-DTPA 0.1 mmol/kg was used as contrast agent for enhanced MRI when combined with a fat suppression scan technique. We took care to not mistake normal dural enhancement due to the rich vascular supply of the region by looking for the “tram-track sign.” Two senior radiologists reviewed the MRI images as necessary.

Serum was drawn at the Examination Center for Biomedical Research of BFH. Blood samples were collected as part of routine treatment for this study.

### Statistical Analysis

Cohort differences in age and visual acuity were analyzed using the Mann-Whitney test, Pearson χ^2^ tests, or Fisher's exact test were used to control for gender, clinical symptoms, prognosis, and bilateral involvement. All statistical analyses were performed using Statistical Package for the Social Sciences software V.19.0 (IBM Corporation). Statistical significance was defined as *p* < 0.05.

## Results

### Demographics and Clinical Characteristics

**Figure 1:**
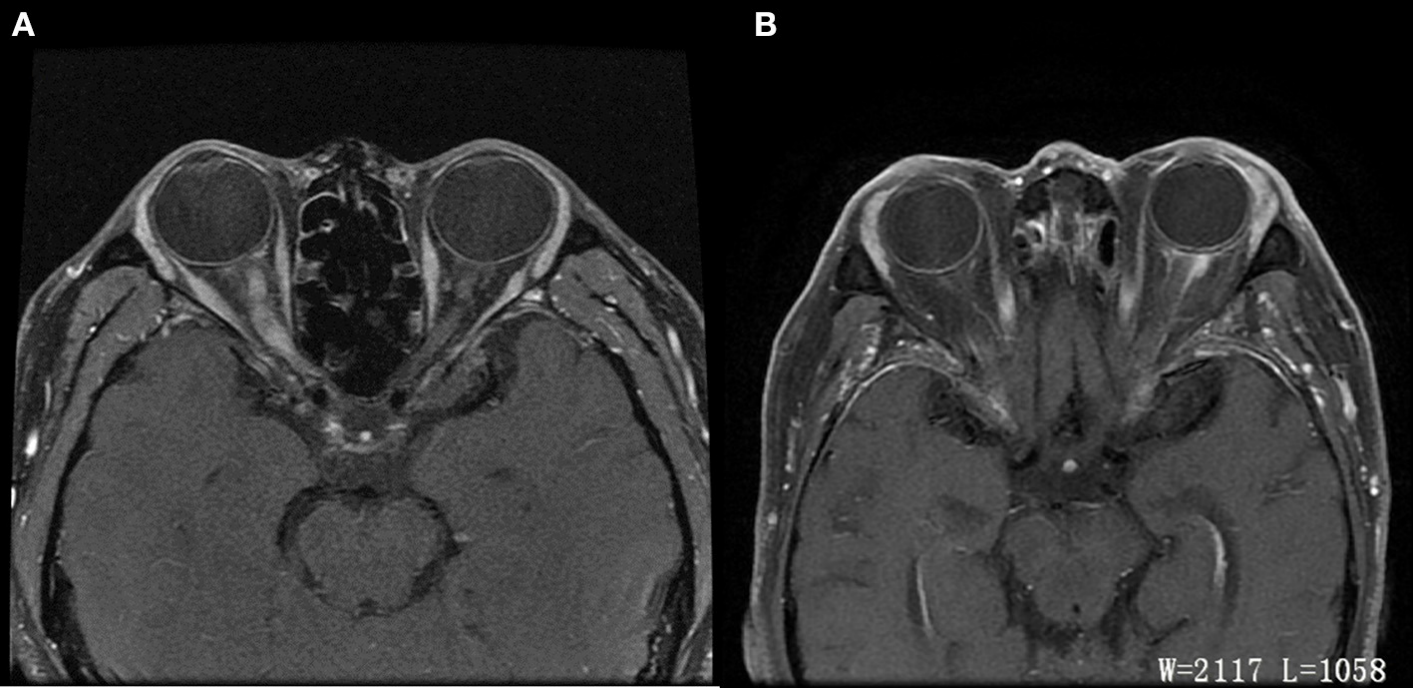
Differences of ON and OPN in MRI. **(A)** ON: Axial T1 contrast image showed enhancement of right optic nerve. **(B)** OPN: Axial T1 contrast image showed enhancement of left optic sheath, not enhancement of optic nerve.

Forty-four patients (23 males and 21 females) had unilateral or bilateral OPN which included a total of 74 eyes. Age at OPN diagnosis ranged from 26 to 64 years (mean ± *SD*: 52.64 ± 17.42). A summary of the demographics of our population can be found in Table 1. Notable ophthalmologic findings include 11 patients with ptosis, nine patients with diplopia, and 10 patients with exophthalmos. Visual field abnormalities included irregular defects 27 (52.94%), peripheral island defects 16 (31.37%), and diffuse visual field defect 8 (15.69%). The optic disc was swollen in 55 eyes and MRI showed enhancement of the optic nerve sheath in all cases (Figure 1).

**Table 1:** OPN patient demographics and clinical characteristics.

Number of patients (*n*)	44
Number of eyes associated with disease (*n*)	74
Median age, years (mean ±*SD*)	52.64 ± 17.42
Eyes with vision loss (*n*)	68
Eyes with papilledema (*n*)	55
Ocular pain *n* (%)	24 (54.55%)
Ptosis *n* (%)	11 (14.86%)
Diplopia *n* (%)	9 (20/45%)
Exophthalmos *n* (%)	10 (13.51%)
Visual field defect (*n*)	51
Central scotoma *n* (%)	0 (0%)
Bjerrum scotoma *n* (%)	0 (0%)
Ring cotoma *n* (%)	27 (52.94%)
Irregular defect *n* (%)	16 (31.37%)
Diffuse visual field defect *n* (%)	8 (15.69%)
Connective tissue disease (*n*)	33
Graves' disease (*n*)	12
IgG-4 RD (*n*)	10
GAP (*n*)	4
SLE (*n*)	2
Sarcoidosis (*n*)	1
Rheumatoid arthritis (*n*)	1
Scleroderma (*n*)	1
Behcet's disease (*n*)	1
Gout (*n*)	1
Infectious diseases (*n*)	10
Tuberculosis (*n*)	5
Sparganosis (*n*)	1
Ocular toxoplasmosis (*n*)	1
Bacterial meningitis (*n*)	1
Fungal meningitis (*n*)	1
Cytomegalovirus (*n*)	1
Idiopathic disease (n)	1

*GAP, Granulomatosis with Polyangiitis; SLE, systemic lupus erythematosus; IgG-4 RD, immunoglobulin G4-related disease*.

### OPN Etiologies

Special autoimmune diseases and infectious diseases were the main causes of the OPN. In line with previous reports, we OPN in patients with Graves' diseases (12), IgG-4 (10), GPA (4), SLE (2), sarcoidosis (1), Rheumatoid arthritis (1), scleroderma (1), Behcet's disease (1), gout (1), tuberculosis (5), sparganosis (1), ocular toxoplasmosis (1), bacterial meningitis (1), fungal meningitis (1), and cytomegalovirus (1), as well as one patient diagnosed with idiopathic inflammation (Table 1).

### Differences Between OPN and IDON

We compared OPN and IDON in Table 2. OPN was more often associated with CTD and infection, IDON was more likely to be associated with MS and NMO (*p* = 0.04). OPN was bilateral in 26 (78.79%) patients compared to 17 (27.87%) patients with (*p* < 0.001). Visual impairment in OPN was also less severe compared to IDON, as LogMAR visual acuity scores of patients with OPN 0.55 ± 0.91 in OPN patients vs. 1.19 ± 1.24 in IDON patients (*p* < 0.001). Eleven (33.33%) OPN patients had ptosis in addition to nine (27.27%) with diplopia and 10 (30.30%) with exophthalmos yet none of the INOD patients had these ophthalmologic findings.

**Table 2:** Clinical features of patients with OPN with autoimmune diseases vs. optic neuritis.

	**OPN**	**ON**	***p***
Number of patients (*n*)	33	61	
Number of eyes associated with disease (*n*)	59	78	
Sex ratio (M/F)	19/14	25/36	0.124
Median age, years (mean ±*SD*)	54.21 ± 17.10	44.56 ± 14.59	0.004^**^
Bilateral involvement, *n* (%)	26 (78.79%)	17 (27.87%)	0.000^**^
Acute onset, *n* (%)	12 (36.36%)	61 (100%)	0.000^**^
Visual Acuity, (LogMar score)	0.55 ± 0.91	1.19 ± 1.24	0.000^**^
Ptosis, *n* (%)	11 (33.33%)	0 (0%)	0.000^**^^#^
Diplopia, *n* (%)	9 (27.27%)	0 (0%)	0.000^**^^#^
Exophthalmos, *n* (%)	10 (30.30%)	0 (0%)	0.000^**^^#^
Connective tissue disease, *n* (%)	33 (100%)	7 (11.48%)	0.000^**^^#^
MS	0 (0%)	8 (13.11%)	0.040^#^
NMOSD	0 (0%)	7 (11.48%)	0.040^*^^#^

*MS, multiple sclerosis; NMOSD, neuromyelitis optica spectrum disorders*.

* *p < −0.05*,

** *p < 0.01*,

# *isher's exact test*.

### OPN and Autoimmune Diseases

**Figure 2:**
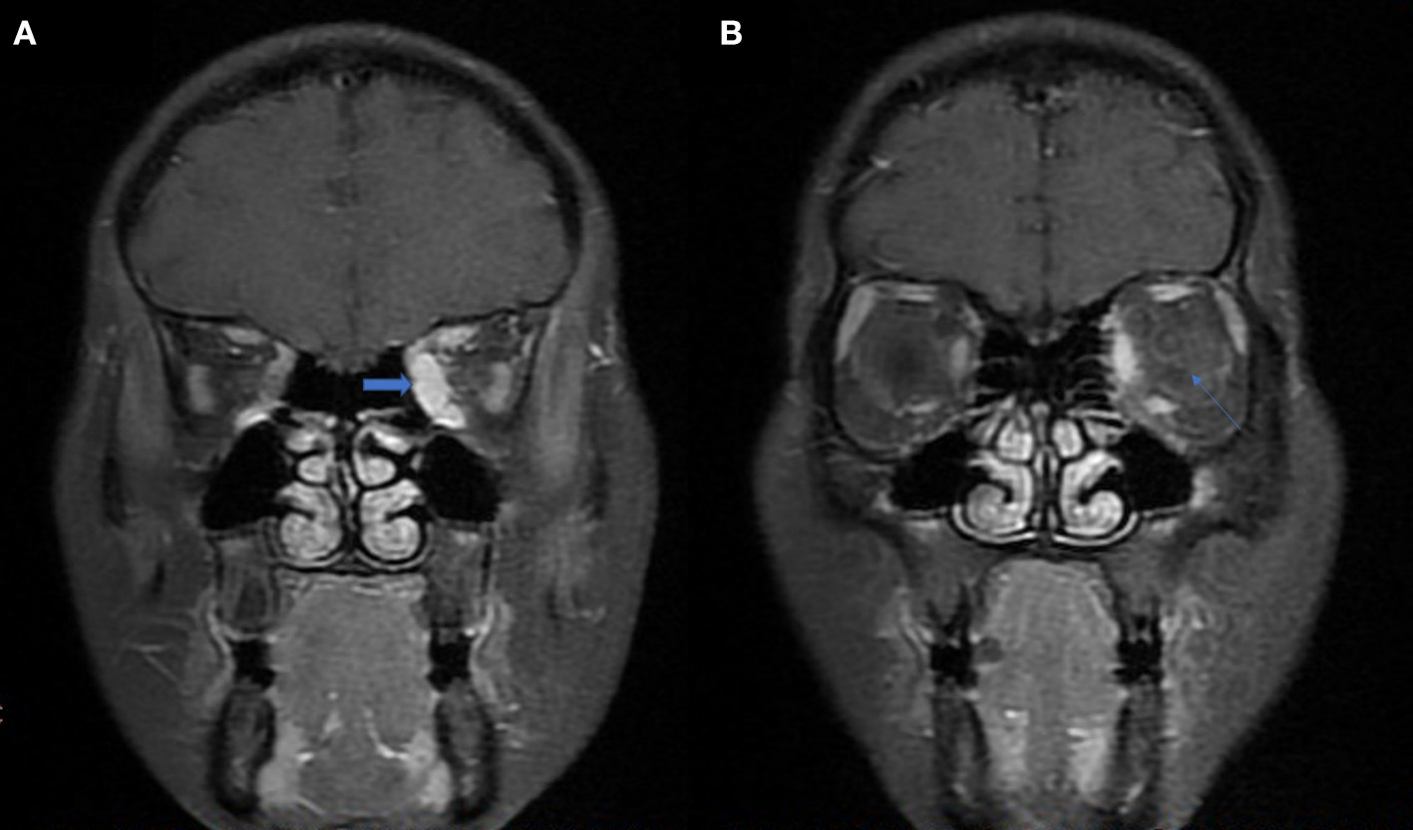
MRI findings of OPN patients with Graves' disease. **(A)** Coronal contrast enhanced T1-weighted MRI showing extraocular muscle belly enlargement (arrow). **(B)** Coronal contrast enhanced T1-weighted MRI showing thickening and enhancement of the left optic nerve sheath (arrow).

**Figure 3:**
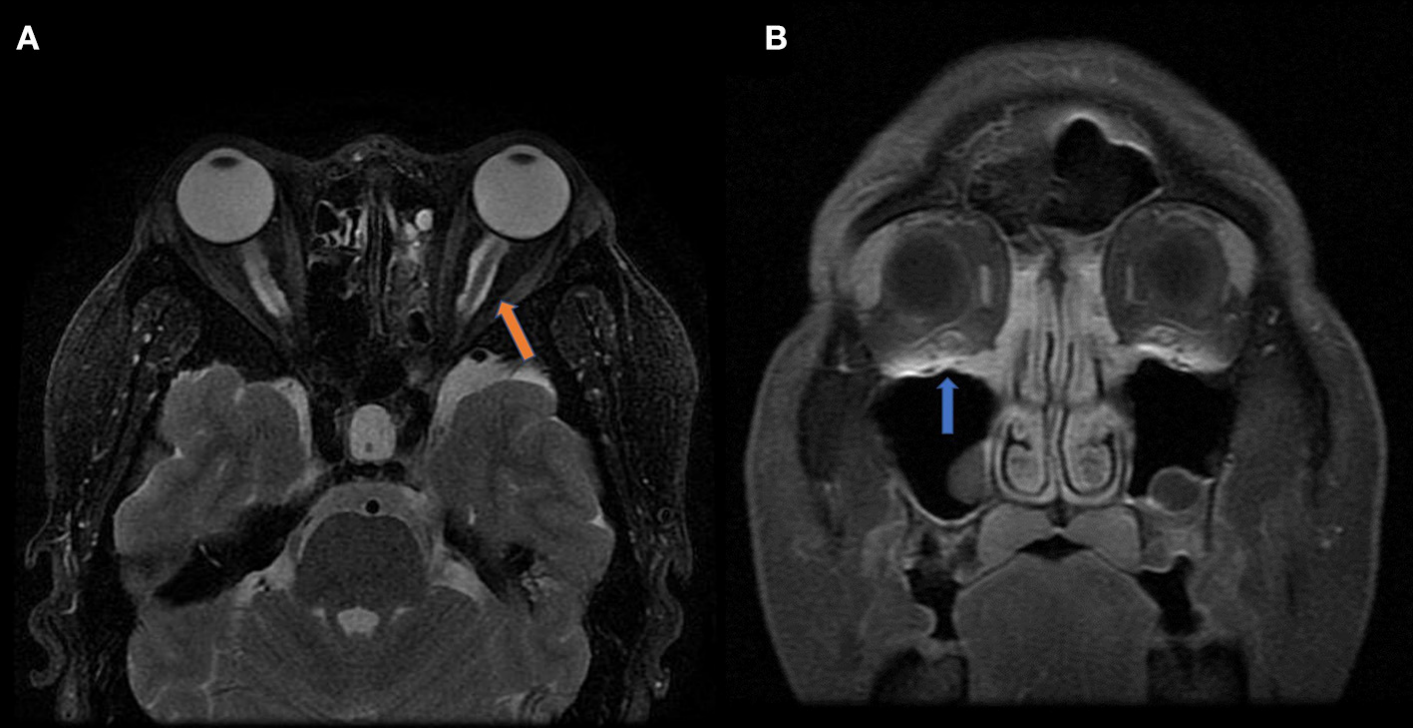
MRI findings of OPN patients with IgG-4 RD. **(A)** Coronal T2-weighted MRI showing swelling and high signal of the bilateral optic nerve sheath (arrow). **(B)** Coronal contrast enhanced T1-weighted MRI showing bilateral infraorbital nerve enlargement (arrow).

**Figure 4:**
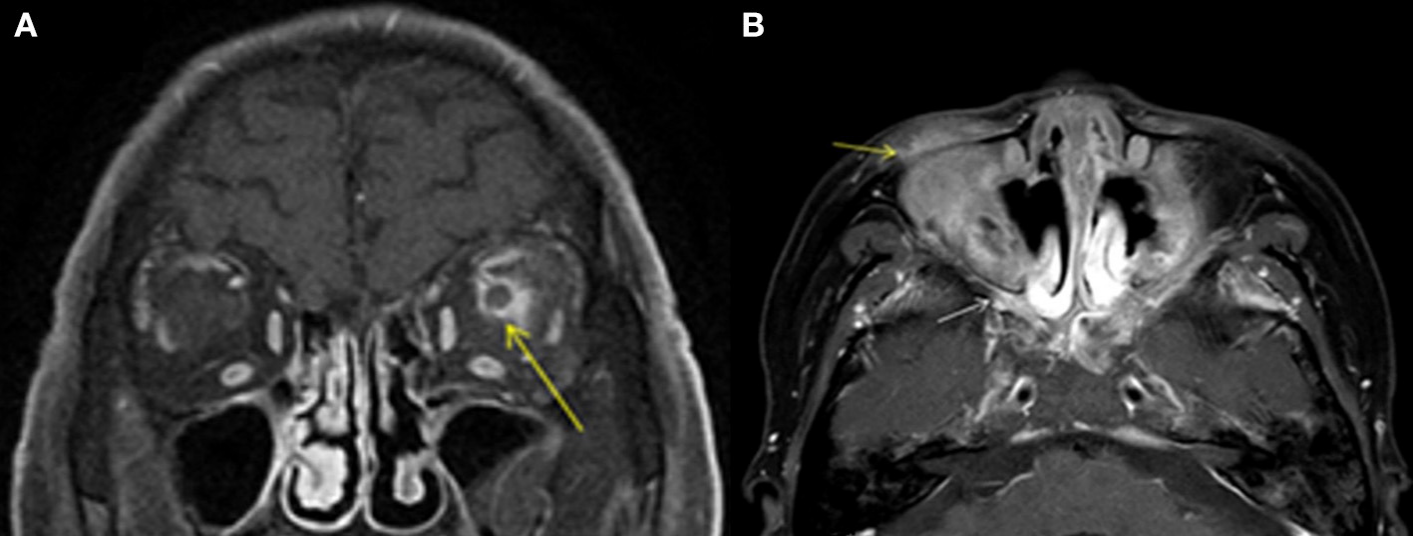
MRI findings of OPN patients with GAP. **(A)** Coronal contrast enhanced T1-weighted MRI showing thickening and enhancement of the left optic nerve sheath (arrow). **(B)** Axial contrast enhanced T1-weighted MRI showing abnormal contrast enhancement shadow in the bilateral pterygopalatine fossa (white arrow) and inflammatory infiltration in the eyelid (yellow arrow).

**Figure 5:**
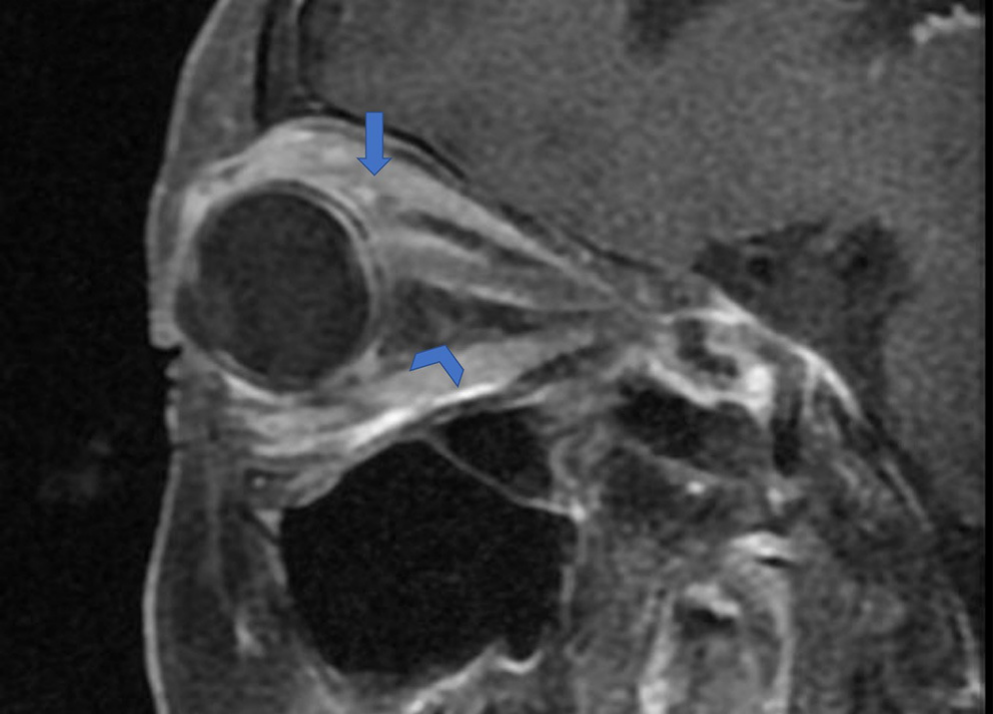
Orbital fat inflammation and inflammatory cell infiltration in OPN. Sagittal contrast enhanced T1-weighted MRI showing orbit fat infiltration (arrowhead) and abnormal contrast enhancement on attachment point of the extraocular muscle (arrow).

Graves' disease, IgG-4, and GPA were the major causes of OPN. Common clinical symptoms of OPN are ocular pain, decreased vision, and visual field defects and orbital MRI with abnormal enhancement around the optic nerve sheath (Table 3). However, OPN characteristics of patients with these special autoimmune diseases were different (Table 4). The most prominent feature of thyroid-associated ophthalmopathy (Graves' disease) was the thickening and protruding of extraocular muscles (Figure 2). Characteristic ophthalmologic findings in OPN patients with IgG-4 involved ocular herniation with involvement of trigeminal nerve branches, resulting in nerve thickening (Figure 3). In GPA patients, formation of an orbital inflammatory pseudotumor was found in the external space of the muscle cone, especially in the inner inferior quadrant (Figure 4). Soft tissue inflammation in the orbit was found in OPNs associated with CTDs along with otherwise abnormal contrast enhancement on the attachment point of the extraocular muscle (Figure 5). This suggests that inflammation of the soft tissue extending to optic nerve sheath may be part of the pathogenesis of OPN which could be due to increased vascular permeability, inflammatory cell leakage, and tissue edema.

**Table 3:** Neuro-ophthalmic findings of OPNs with autoimmune diseases.

**Diseases**	**Cases (*n*)**	**Age (year)**	**Sex (*n*, male/female)**	**Imaging**	**Ophthalmic involvement (*n*)**	**Unilateral or bilateral involvement (*n*)**	**Treatment**	**Patients of recurrence (*n*)**
Graves' disease	12	32–77	7/5	Orbit MRI	Eye Pain: Vision Loss: 10 Visual field defect: 11 Optic papilledema: 5	Unilateral: 3 Bilateral: 9	Glucocorticoid	3
IgG-4	10	39–80	7/3	Orbit MRI	Eye Pain: 7 Vision Loss: 7 Visual field defect: 9 Optic papilledema: 5	Unilateral: 1 Bilateral: 9	Glucocorticoid	2
GAP	4	23–72	3/1	Orbit MRI	Eye Pain: 3 Vision Loss: 4 Visual field defect: 3 Optic papilledema: 4	Unilateral: 1 Bilateral: 3	Glucocorticoid	2
SLE	2	29–58	0/2	Orbit MRI	Eye Pain: 0 Vision Loss: 2 Visual field defect: 1 Optic papilledema: 2	Bilateral: 2	Glucocorticoid	2
Sarcoidosis	1	60	0/1	Orbit MRI	Eye Pain: 1 Vision Loss: 1 Visual field defect: 1 Optic papilledema: 1	Bilateral: 1	Glucocorticoid	0
Rheumatoid arthritis	1	63	1/0	Orbit MRI	Eye Pain: 1 Vision Loss: 1 Visual field defect: 1 Optic papilledema: 1	Bilateral: 1	Glucocorticoid	1
Scleroderma	1	72	0/1	Orbit MRI	Eye Pain: 1 Vision Loss: 1 Visual field defect: 1 Optic papilledema: 1	Unilateral: 1	Glucocorticoid	1
Behcet's disease	1	15	0/1	Orbit MRI	Eye Pain: 1 Vision Loss: 1 Visual field defect: 1 Optic papilledema: 1	Unilateral: 1	Glucocorticoid	1
Gout	1	36	1/0	Orbit MRI	Eye Pain: 0 Vision Loss:1 Visual field defect: 1 Optic papilledema: 1	Unilateral: 1	Glucocorticoid	1

*IgG-4, Immunoglobulin G4-related disease; GAP, granulomatosis with polyangiitis; SLE, systemic lupus erythematosus; MRI, magnetic resonance imaging*.

**Table 4:** Orbital MRI findings of OPNs with CTD.

**Orbital MRI finding**	**Graves' disease (*n* = 12)**	**IgG-4 (*n* = 10)**	**GAP (*n* = 4)**	**SLE (*n* = 2)**	**Sarcoidosis (*n* = 1)**	**Rheumatoid disease (*n* = 1)**	**Scleroderma (*n* = 1)**	**Behcet's disease (*n* = 1)**	**Gout (*n* = 1)**
Abnormal contrast enhancement surrounding the intraorbital optic nerve	12	10	4	2	1	1	1	1	1
Orbit fat infiltration	12	10	4	2	1	1	1	1	1
Myositis	12	5	4	0	1	0	0	0	0
Trigeminal nerve branch involvement	0	9	0	0	0	0	0	0	0
Pterygopalatine fossa pseudotumor	0	0	4	0	0	0	0	0	0
Dacryoadenitis	10	10	4	0	1	0	0	0	0
Maxillary sinus disease	1	6	3	0	0	0	0	0	0
Paranasal sinus disease	1	7	4	0	1	0	0	0	0
Otitis mastoidea	0	0	3	0	0	0	0	0	0
Pachymeningitis	0	1	1	0	1	0	0	0	0

*GAP, Granulomatosis with Polyangiitis; SLE, systemic lupus erythematosus; IgG-4 RD, immunoglobulin G4-related disease*.

## Discussion

OPN is an uncommon orbit inflammatory disease (7) and most prior literature has come in the form of case reports (8). A cohort of 14 patients with idiopathic OPN was reported in 2001 (1). This study identified demographic and clinical characteristics of patients with OPN, which included mostly middle-aged females with unilateral presentation, +/− eye pain, mild visual impairment, and optic disc edema. Visual field abnormalities included arcuate defects, paracentral scotomas, central scotomas, and peripheral island defects (3). The clinical manifestations and characteristics of the patients in our study are quite different. This may be due to the differing OPN etiologies, as many of the patients in this small cohort had idiopathic OPN not associated with another specific condition.

It has been previously established that OPN is an orbital inflammatory disease that is different from IDON (9). Our study illustrates some of the differences between OPN and IDON, which include clinical characteristics, imaging features, and prognosis. We found that OPN are more often with autoimmune diseases and IDON are more often associated with idiopathic inflammation, such as MS and NMO. Orbital MRI showed low or equal signal on T1WI and slightly low signal on T2WI. In OPN, contrast-enhanced scans showed that most of the optic nerve sheath presented a strip-like or nodular uniform enhancement with unclear boundaries. Optic nerve and sheath enhancement were present in IDON. Soft tissue inflammation in the orbit has also been found in OPN, yet inflammation of the orbit is rare in IDON. Unlike OPN, optic nerve sheath enhancement in IDON is almost always accompanied by more prominent optic nerve parenchyma enhancement. And we summary clinical the radiological features of OPN in the Table 5.

**Table 5:** Clinical and radiological features of optic perineuritis.

**Age**	**Middle-aged**
Sex	1:1
Combined with systemic diseases	Autoimmune diseases
Bilateral	+
Eye Pain	+/−
Vision Loss	Mild to moderate
Visual field defect	peripheral island defects
Fundus manifestations	Optic papilledema
Radiological features	Contrast enhancement surrounding the intra-orbital optic nerve
Glucocorticoid	Effective
Prognosis	Good

Known autoimmune diseases etiologies of OPN include sarcoidosis (10, 11), GAP (12, 13), giant cell arteritis (14, 15), Crohn's disease (16), IgG-4 (17), Behcet's disease (9), and SLE (2). Infectious etiologies of OPN have been associated with acute retinal necrosis (18), syphilis (19, 20), herpes zoster (21), tuberculosis (22, 23), and Mycoplasma pneumoniae (24). In our study we found the primary causes of OPN were Graves' diseases (12), IgG-4 (10), GPA (4), SLE (2), sarcoidosis (1), Rheumatoid arthritis(1), scleroderma (1), Behcet's disease (1), gout (1), tuberculosis (5), sparganosis (1), ocular Toxoplasmosis (1), bacterial meningitis (1), fungal meningitis (1), and Cytomegalovirus (1). Unlike published reports, we did not find and OPN associated with syphilis and giant cell arteritis, which may be due to our small sample size and single center study. The finding of OPN associated with Graves' disease is unique to our study. We found that orbital fat inflammation was always associated with inflammation of the optic nerve sheath, suggesting that inflammatory invasion of orbital fat may be the primary pathogenic mechanism of OPN in patients with Graves' disease.

In GPA, formation of an orbital inflammatory pseudotumor was mainly concentrated in the external space of the muscle cone, especially in the inner inferior quadrant (25). OPN patients with IgG-4 often had ocular herniation involving the trigeminal nerve branches, which resulted in its thickening, suggesting perineural growth may be a characteristic imaging finding of OPN (26). The most prominent feature of thyroid-associated ophthalmopathy is the thickening and protruding of extraocular muscles (27). The trigeminal nerve branches were not involved in the OPN of the GPA patients in this study and the extraocular muscles were not thickened. We were unable to find any prior reports of trigeminal nerve branching and extraocular muscle involvement in patients with GPA, Graves', and sarcoidosis.

Autoimmune diseases are a group of diseases that affect bones, joints, and surrounding soft tissue, such as muscles, bursa, tendons, fascia, and nerves (28). Chronic inflammation, mediated by T and B lymphocytes, eosinophils, and macrophages, activates fibroblasts to induce collagen deposition, and ultimately leads to tissue proliferation and dural thickening (29). The optic nerve is a continuation of the central nervous system with a dura, arachnoid, and pia mater. Therefore, the optic nerve sheath is also susceptible autoimmune diseases. Orbital MRI showed low or equal signal on T1WI and slightly low signal on T2WI. Contrast-enhanced scanning showed that most of the optic nerve sheath had strip-like or nodular uniform enhancement with unclear boundaries. Most cases of soft tissue inflammation in the orbit have been found in OPN, differing it from the imaging findings in IDON. We speculate that OPN may be caused by orbital soft tissue inflammation and that the damage of the optic nerve sheath could be from antigen/antibody-mediated destruction associated with rheumatoid arthritis.

In recent studies, some cases which had been diagnosed with idiopathic optic perineuritis were found that were associated with NMOSD (30) and antibody against myelin oligodendrocyte glycoprotein (MOG-Ab) (31, 32). A Korea study showed that compare to the optic neuritis with MOG-Ab, ocular pain with ocular movement and optic disc swelling were more common in patients with optic nerve sheath enhancement (ONSE), who also exhibited a poorer initial visual acuity than did those without ONSE (33). In our studies, there was one OPN patient without any autoimmune and infection diseases. However, MOG-Ab testing were not performed, we also think it should be performed in the patients with idiopathic OPN.

Limitations of this study include its retrospective design and recruitment of patients form only one medical center. Only 44 cases were included in this study due to inability to follow-up with some patients who initially met the inclusion criteria. Multi-center, large sample, case-control prospective studies are needed to further understand OPN pathogenesis and its relationship with other diseases.

## Conclusions

OPN is often associated with special autoimmune diseases such as Graves' disease, GPA, and IgG-4 disease. MRI findings of OPN patients included orbital fat inflammation, pachymeningitis, inflammation of optic sheath vessels, and edema of the optic nerve sheath which may provide insights into OPN pathogenesis. The possibility of OPN should be considered in the differential diagnosis of atypical optic neuritis to avoid misdiagnosis as IDON or other similar ophthalmologic pathologies discussed in this study.

All authors read and approved the final version of the manuscript.

## Data Availability Statement

The raw data supporting the conclusions of this article will be made available by the authors, without undue reservation.

## Ethics Statement

This study was approved by the BFH Ethics Committee and was conducted following the latest iteration of the Declaration of Helsinki (version: 2019-P2-201-01). Participants were given written informed consent (version V1.1/2019-09-16) before inclusion in the study. Written informed consent was obtained from the individual(s) for the publication of any potentially identifiable images or data included in this article.

## Author Contributions

HL and JL was involved in the conception and design of the study. HW and ZW reviewed the orbit MRI images. JS and JL contributed to the acquisition, analysis, and interpretation of data as well as drafting the manuscript and revising it critically. HZ and YW were the duty of diagnosis the disease. JL has also provided final approval of the version to be published. All authors have given final approval of the version to be published and agreed to be accountable for all aspects of the work in ensuring that questions related to the accuracy or integrity of any part of the work are appropriately investigated and resolved.

## Conflict of Interest

The authors declare that the research was conducted in the absence of any commercial or financial relationships that could be construed as a potential conflict of interest.

## Abbreviations

OPN: optic perineuritis
NMOSDs: optica spectrum disorders
MS: multiple sclerosis
ON: optic neuritis
MRI: magnetic resonance imaging
IDON: idiopathic demyelination optic neuritis
CTD: connective tissue disease
IgG-4 RD: Immunoglobulin G4-related disease
GAP: granulomatosis with polyangiitis
SLE: systemic lupus erythematosus
ONS: optic nerve sheath
OCT: Optical coherence tomography
BFH: Beijing Friendship Hospital
MOG: Anti-myelin oligodendrocyte glycoprotein.
